# Potential Use of Wastewater Treatment Plant Washed Mineral Waste as Flood Embankment Materials

**DOI:** 10.3390/ma18143384

**Published:** 2025-07-18

**Authors:** Jacek Kostrzewa, Łukasz Kaczmarek, Jan Bogacki, Agnieszka Dąbska, Małgorzata Wojtkowska, Paweł Popielski

**Affiliations:** 1 Department of Hydro-Engineering and Hydraulics, Faculty of Environmental Engineering, Warsaw University of Technology, Nowowiejska 20, 00-653 Warsaw, Poland; lukasz.kaczmarek@pw.edu.pl (Ł.K.); agnieszka.dabska@pw.edu.pl (A.D.); pawel.popielski@pw.edu.pl (P.P.); 2 Department of Informatics and Environmental Quality Research, Faculty of Environmental Engineering, Warsaw University of Technology, Nowowiejska 20, 00-653 Warsaw, Poland; jan.bogacki@pw.edu.pl (J.B.); malgorzata.wojtkowska@pw.edu.pl (M.W.)

**Keywords:** washed mineral waste, grit chamber, wastewater treatment plant, geotechnical properties, heavy metal immobilization level, flood embankments, construction materials, circular economy

## Abstract

Recycling washed mineral waste, generated as a byproduct of the mechanical wastewater treatment process, can be a beneficial alternative to widely used natural sand in construction. Studies on material from the Warsaw agglomeration, available in quantities sufficient for construction applications, demonstrated its high usability in specific hydrotechnical applications. Key laboratory tests for material characterization included physical, permeability, mechanical, and chemical property analyses. The tested waste corresponds to uniformly graded medium sands (uniformity coefficient: 2.20) and weakly calcareous (calcium carbonate content: 2.25–3.29%) mineral soils with organic content ranging from 0.24% to 1.49%. The minimum heavy metal immobilization level reached 91.45%. At maximum dry density of the soil skeleton (1.78/1.79 g/cm^3^) and optimal moisture content (11.34/11.95%), the hydraulic conductivity reached 4.38/7.71 m/d. The mechanical parameters of washed mineral waste included internal friction angle (34.4/37.8°) and apparent cohesion (9.37/14.98 kPa). The values of the determined parameters are comparable to those of natural sands used as construction aggregates. As a result, washed mineral waste has a high potential for use as an alternative material to natural sand in the analyzed hydrotechnical applications, particularly for flood embankment construction, by applicable technical standards and construction guidelines.

## 1. Introduction

The circular economy (CE) is a concept of sustainable economic development based on shifting from a linear resource utilization paradigm, following the ’take–make–dispose‘ principle, to a circular model. In this model, waste, if generated, becomes a valuable resource. This concept has gained popularity since the 1970s, with its formulation attributed to [1]. The goal of the CE is to promote responsible and efficient resource utilization, including mineral raw materials, while maximizing secondary resource recovery and waste recycling to replace materials extracted from natural resources [2,3,4]. This approach should align with global development strategies, particularly with Goal 12, ‘Responsible Consumption and Production’, of the 2030 Agenda for Sustainable Development [5]. Findings by [6] confirm the growing interest in studying circular economy policies regarding the environment. The CE has also become one of the key strategic directions for the development of the European Union’s economy since the European Commission introduced the first CE action plan, ‘Closing the Loop’, in 2015 [7], followed by the second action plan, ‘For a Cleaner and More Competitive Europe’ [8]. The foundation of these economic strategies is sustainable development, which integrates economic, environmental, and social measures to safeguard present and future generations [4,9].

One of the key and widely exploited natural resources is sand [10,11,12,13,14,15,16]. It is a fundamental material for the economy, representing an enormous volume of extracted solid material, particularly for the construction sector, which is the biggest consumer of global sand resources [17,18]. Sand has many applications, including in road embankments and as a key component in concrete, asphalt, glass, and solar panel production. Its popularity in construction stems from its low cost, versatile properties, and ease of extraction. However, the enormous rate of exploitation of individual deposits, as observed in many regions of Poland [19], exceeds its natural renewal rate, making access to this resource (including rising transportation costs) an increasingly critical barrier to development [20]. The negative impact of uncontrolled or even predatory extraction of global sand resources on socio-political, economic, and primarily environmental conditions is widely discussed in the literature [10,11,12,13,15,16,18,21,22,23,24,25,26,27]. The identified issues include the lack of international strategies regulating sand extraction, use, and trade; underreporting or absence of reliable statistics on sand mining and consumption; international disputes and domestic shortages of sand resources; the rise of organized crime engaged in illegal sand mining and trade; threats to the lives of individuals trying to prevent uncontrolled extraction; increasing censorship of media and universities; destruction of nature reserves and protected areas; changes in coastal ecosystems (including river, coastline, and beach erosion, leading to the undermining of engineering structures such as bridges, retaining walls, and water supply facilities); lowering of groundwater levels; intensification of extreme events (such as floods, droughts, and storms); impacts on drinking water supplies (river pollution and pH level changes); reduction in food source productivity (including threats to freshwater and marine fisheries); threats to biodiversity, including endangered species; and epidemiological risks to local populations.

Due to the significant demand for sand aggregates in construction, improving the sustainability of global sand resource management requires a thorough selection process for aggregates based on their application, location, and intended use in construction projects. In line with the CE concept, replacing natural sand with alternative materials holds substantial potential across various construction applications. This shift could lead to notable conservation of natural sand resources for other purposes, reducing the costs associated with extraction, processing, and transportation, and, ultimately, lowering construction, modernization, and renovation expenses for structures utilizing alternative materials [28,29,30,31]. Examples of such solutions include recycling waste generated in wastewater treatment plants [32,33,34,35,36,37], with one innovative alternative raw material being sand recovered from mineral waste produced during mechanical wastewater treatment. There are two main categories of mineral waste. The first consists of untreated mineral waste (MW), with waste code 19 08 02 according to the European Waste Catalogue established in 2014 [38], obtained directly from grit chambers. The second one includes washed mineral waste (WMW), with waste code 19 12 09 according to the European Waste Catalogue [38], received from the rinsing process of mineral waste from grit chambers. The existing literature on sand recovered from mineral waste produced in wastewater treatment plants and suggested applications based on laboratory tests is presented in Table 1.

The most frequently determined tests of mineral waste included the content of organic substances and particle size distribution.

The content of organic substances in untreated mineral waste can reach up to 68.49% [45]. Even though the authors [39,42] indicated that sand was recovered from untreated mineral waste (waste code 19 08 02), it can be concluded that this waste was nevertheless subjected to a rinsing process in the wastewater treatment plant. The content of organic substances found in the waste samples did not exceed 2% (1.11% and 1.80%, respectively), which indicates effective rinsing of this type of matter. Moreover, in the case of the wastewater treatment plant in Cracow [42], the authors suggest renovating the plant by installing washing separators, which ensure the rinsing and separation of organic parts from the mineral sand pulp [40]. On the other hand, the article [43] presents the results of tests on samples of washed mineral waste with different degrees of rinsing. The content of organic substances in the best-washed waste (with a moisture content of 2.64%) was 0.78%, while the least-washed waste (with a moisture content of 19.98%) contained 14.44% of organic substances.

The authors [19,37,41,42,43,44] unanimously emphasize that the possibility of using mineral waste in construction requires careful rinsing, among others, to minimize organic matter. As indicated by [44] in their research, cleaning and drying mineral waste from grit chambers is effective and reduces organic substances content by up to 67.1% (while decreasing moisture by 98.8%).

The dominant fraction of both categories of mineral waste is the sand fraction, which usually exceeds 90% of their mass [40,41,42,43,44]. Therefore, they are called sandy waste, and their properties are comparable to soils or construction aggregates [19]. Moreover, they can be classified as medium sand with small amounts of gravel [40,42,43]. Based on the uniformity and curvature coefficient results, they can be considered as uniform-grained (mono-fractional) materials. The uniformity coefficient reaches values of 0.89–2.20, while the curvature coefficient is 0.91–1.02 [40,42,45].

Additionally, taking into account the amount of mineral waste generated (3156 tons/year) in a wastewater treatment plant with a capacity of 165,000 m^3^/day and the costs of its disposal (PLN 2000/ton) [42], the practical application of this waste in the construction industry has an additional advantage to the wastewater treatment plants themselves.

The article aims to present the physical, permeability, mechanical, and chemical properties of washed mineral waste (WMW), along with an assessment of their potential use as materials for the construction of flood embankments. The application of waste as a construction material intended for hydro embankments, which permanently or periodically retain water, necessitates a comprehensive characterization of the parameters of this waste. An extensive series of laboratory physical tests were performed, which included granulometric composition (fraction content), specific density, quantities characterizing the limiting states of compaction (dry density, void ratio, and porosity corresponding to the state of the loosest and densest possible composition of soil grains), maximum dry density, optimal moisture content, and degree of saturation after compaction. The hydraulic conductivity characterized the permeability tests. As part of the mechanical tests, direct and triaxial shear tests, as well as oedometric modulus tests, were conducted. The content of organic substances, calcium carbonates, pH, specific electrical conductivity, and heavy metal content (zinc, lead, copper, cadmium, chromium, nickel, and cobalt) in wastes and their extracts, and consequently, the determination of the degree of immobilization of heavy metals, were subject to chemical tests.

The presented scope of research, allowing for a comprehensive and precise comparative analysis of the properties of WMW over the years, represents an innovative approach enabling the adaptation of waste with specific characteristics to particular construction applications, including formulating suitability criteria. The generation of waste with a certain degree of similarity across wastewater treatment plants suggests that the studies, aspects, and practical application proposals discussed in the article may be of significant interest to scientists, technologists, and engineers in wastewater treatment plants worldwide. In the international literature, there is a lack of information on studies of the properties of WMW from wastewater treatment plants that would indicate the possibility of its use as a material for the construction of flood embankments.

## 2. Materials and Methods

Samples of WMW were collected from a wastewater treatment plant designed for 2,100,000 PE (population equivalent, according to [46]) from a temporary storage site located in an open area. Four samples (W1, W2, W3, and W4) were taken in March 2021. These samples were mixed in equal mass proportions to create an additional sample (W1.4). The last sample (P-2) was collected in November 2023. The article primarily focuses on presenting the test results for samples W1.4 and P-2 and supplementary test results for the previously analyzed sample W4, which was examined in an earlier study. The methods used in soil testing were applied to determine the properties of WMW.

In the first stage, the samples were dried to a constant mass at 105–110 °C in an electric laboratory dryer and then cooled.

The granulometric composition (fraction content) was examined using the sieve analysis method (described by [47]) on an electromechanical shaker following the standard [48]. For this purpose, a set of sieves with mesh sizes of 4, 2, 1, 0.5, 0.25, 0.125 or 0.1, and 0.063 mm and a collection pan were used.

A pycnometer was used to determine the specific density. Pisarczyk [49] describes the method. It is a modified version of the test method presented in the standard [50].

The determination of quantities characterizing the limiting states of compaction (dry density corresponding to the state of the loosest and densest possible composition of soil grains) was carried out using a cylinder with a piston and vibrating forks. The test was conducted according to the standard [51].

The determination of the maximum dry density and the optimal moisture content was carried out using a Proctor electromechanical apparatus, following method I (described by [52,53]), following the standard [51]. The waste samples were placed in a small cylinder with a capacity of 1 dm^3^ in three layers, each compacted with 25 blows of a light rammer weighing 2.5 kg, dropped from a height of 32 cm, which corresponded to a unit compaction energy of 0.59 J/cm^3^ of waste. The hydraulic conductivity, shear strength, and compressibility tests were conducted on samples compacted to the parameters obtained during the compaction test in the Proctor apparatus.

The hydraulic conductivity tests were conducted under uniaxial strain conditions using the ITB-ZW-K2 hydraulic apparatus and under triaxial strain conditions using a triaxial compression electromechanical apparatus equipped with two hydraulic pumps, allowing for water pressure control in the upper and lower parts of the sample. The tests in the ITB-ZW-K2 apparatus were performed according to the standard [54] under vertical load, with an upward flow and a constant hydraulic gradient ranging from 0.54 to 0.68. These values fall within the recommended hydraulic gradient range (0.3–0.8) for this type of test, as [55] suggested. For the triaxial apparatus, the tests were conducted following the methods outlined by [56,57] and the standard [58], with an effective confining stress of σ_3_′ = 50 kPa, a downward flow, and a constant hydraulic gradient (i = 1). The results were compared with hydraulic conductivity values determined based on empirical formulas—Hazen, USBR, and Slichter [59], for which the WMW met the applicability range. Furthermore, these calculations utilized the parameters of the tested waste established in the article, including the uniformity coefficient, equivalent diameters (D_10_ and D_20_), and porosity (n).

The mechanical parameters describing shear strength were determined using an electromechanical direct shear apparatus (as described by [60]) and the previously mentioned triaxial compression apparatus. In the first case, a shear box apparatus with a square cross-section and a side length of 8 cm was used to determine shear strength by the standard [61]. Shear strength was measured for five normal stress values (12.5, 25, 50, 100, and 200 kPa). For triaxial tests (isotropically consolidated undrained triaxial test—CIU type), cylindrical samples with a height of 10 cm and a diameter of 5 cm were used, following the methods outlined by [56] and the standard [62]. Shear strength was determined at a shear rate of *v_sh_* = 0.1 mm/min for three values of confining stress (50, 100, and 200 kPa).

The mechanical parameters describing compressibility were determined using mechanical oedometers following the standard [63]. The samples were gradually loaded and unloaded, with each successive load twice as large as the previous one (during loading) or twice as small (during unloading). The applied loads corresponded to those used in the direct shear apparatus test (12.5, 25, 50, 100, and 200 kPa). Load changes in each case were performed after the stabilization of the sample height (as described by [64]).

The content of organic substances and calcium carbonate was determined using Tyurin’s and Scheibler’s methods (as described by [65,66,67]) according to the standards [68,69], respectively.

Waste samples for determining heavy metal content (zinc, lead, copper, cadmium, chromium, nickel, and cobalt) were prepared according to the Titan MPS microwave mineralization methodology. The determination of metal content was performed according to the standard [70] using a PinAAcle 900F spectrometer with the flame atomic absorption spectrometry (FAAS) method, similar to [71,72].

The waste intended for heavy metal content analysis in aqueous extracts was brought into contact with the leaching agent (distilled water) at a liquid-to-solid phase ratio of L/S = 10 L/kg, by the standard [73] (batch test method, as described by [74]). The prepared samples were shaken on a rolling table for 24 h and filtered using a vacuum pump. Similar to other researchers [41,75], in addition to heavy metal content (according to standard [70]), the pH and specific electrical conductivity were also determined (according to the respective standards [76,77]).

The level of heavy metal immobilization was calculated based on Equation (1) presented by [75]:

(1) $$I_{i}=100-\frac{m_{i,e}}{m_{i,m}}\times 100\%,$$

where:

I_i_—the level of immobilization of the i-th heavy metal [%];

m_i,e_—the mass of the i-th heavy metal in the extract [mg];

m_i,m_—the mass of the i-th heavy metal in the material subjected to leaching [mg].

## 3. Results and Discussion

### 3.1. Physical and Permeability Parameters

**Figure 1:**
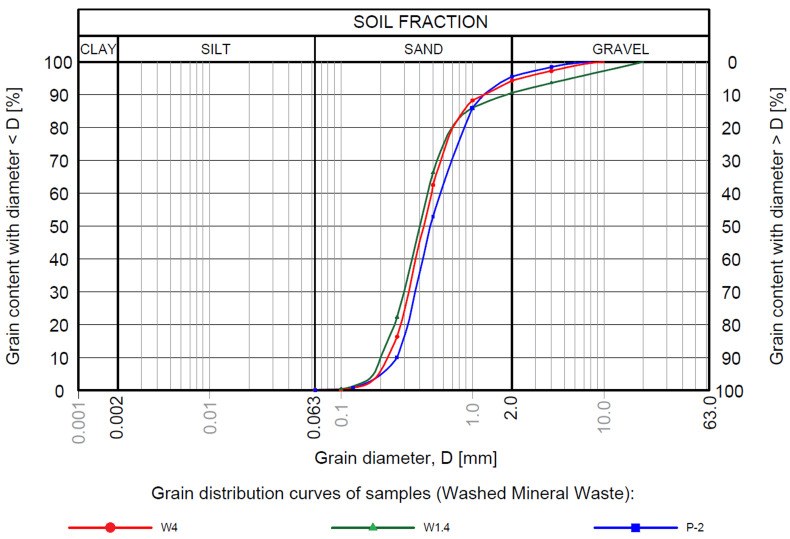
Grain-size distribution curves of washed mineral waste samples according to the European classification [69].

**Figure 2:**
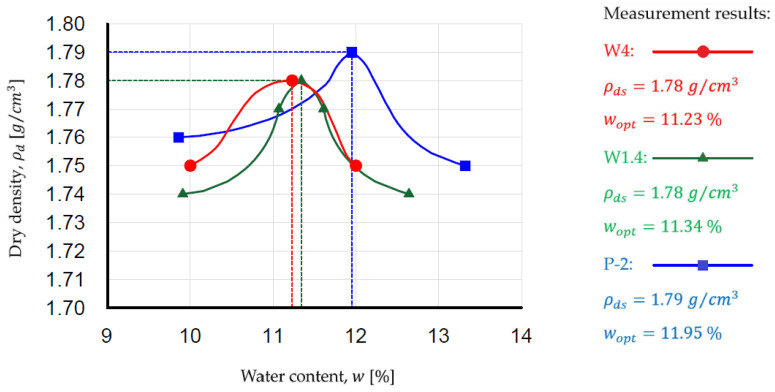
Compaction curves of washed mineral waste.

The results of physical and permeability parameters are presented in Table 2. Grain-size distribution curves according to the European classification [69] and compaction curves of WMW are presented in Figure 1 and Figure 2.

The results of individual physical and permeability parameters of WMW presented in Table 2 are similar for individual samples (W4, W1.4, P-2), indicating the stability of the mechanical wastewater treatment process and, consequently, the production of waste with comparable properties. The laboratory tests of physical parameters (Table 2, nos. 1–20) conducted on WMW confirm the conclusions presented by [40]. The measured parameter values are close to the geotechnical parameter values for sands.

According to the European classification, WMW corresponds to uniformly graded medium sands [78]. The percentage content of individual fractions of WMW presented in the article and their effective diameters and grain-size coefficients are similar to the mineral waste described in previous studies [41,42,43].

The specific density of WMW is similar to the density (2.65 g/cm^3^) of mineral soils with comparable grain-size distribution [60]. Differences in specific density values between the waste and mineral soils may result from organic substances in the waste. It is confirmed that the specific density of mineral waste collected from grit chambers can range from 1.10 to 2.65 g/cm^3^ due to the formation of organic material layers covering mineral particles in wastewater [79]. This statement was also supported by the results of the specific density of mineral waste (average value: 2.55 g/cm^3^) presented by [45]. The obtained specific density values fall within the range (2.30–2.64 g/cm^3^) for humic sands [80].

Considering the specific density of sands (2.65 g/cm^3^) and the range of their void ratios (0.3–1.0) or the porosity of uniformly graded sands (0.258–0.476), the range of dry density values is 1.33–2.04 or 1.39–1.97 g/cm^3^, respectively [55,80]. The results confirm that WMW corresponds to sands, particularly uniformly graded sands, in terms of dry density, void ratios, and porosity.

According to the results of compaction parameter studies for fine-grained non-cohesive soils conducted by [49], the maximum dry density in sands can range from 1.65 to 2.10 g/cm^3^ at an optimum moisture content of 8.0–13.5%, with a degree of saturation corresponding to these parameters in the range of 0.4–0.7. The obtained results from the author’s studies on WMW fall within the specified parameter ranges for sands.

The closest compliance with the flow tests conducted under uniaxial deformation conditions (ITB-ZW-K2 apparatus) was achieved using the Slichter formula. The obtained hydraulic conductivity values fall within the range corresponding to the porosity of the waste. The best match was obtained using the Slichter formula for the minimum porosity value (n_min_) for tests conducted under triaxial stress and deformation conditions and empirical formulas. The hydraulic conductivity values determined in the triaxial compression apparatus were lower than those obtained using the ITB-ZW-K2 apparatus. The empirical formulas by Hazen and USBR, which consider only grain size characteristics (effective diameters and uniformity coefficients), overestimate the laboratory results by two and one orders of magnitude, respectively, compared to ITB-ZW-K2 test results and by three and two orders of magnitude compared to triaxial test results. According to [81], fine and medium sands’ indicative hydraulic conductivity values range from 1–5 m/d and 5–30 m/d, respectively. The results of the ITB-ZW-K2 tests on WMW (P-2) fall within the range for medium sands, while (W1.4) corresponds to fine sands. In the case of triaxial tests, the results align with the typical values for fine sands.

### 3.2. Mechanical Parameters

**Figure 3:**
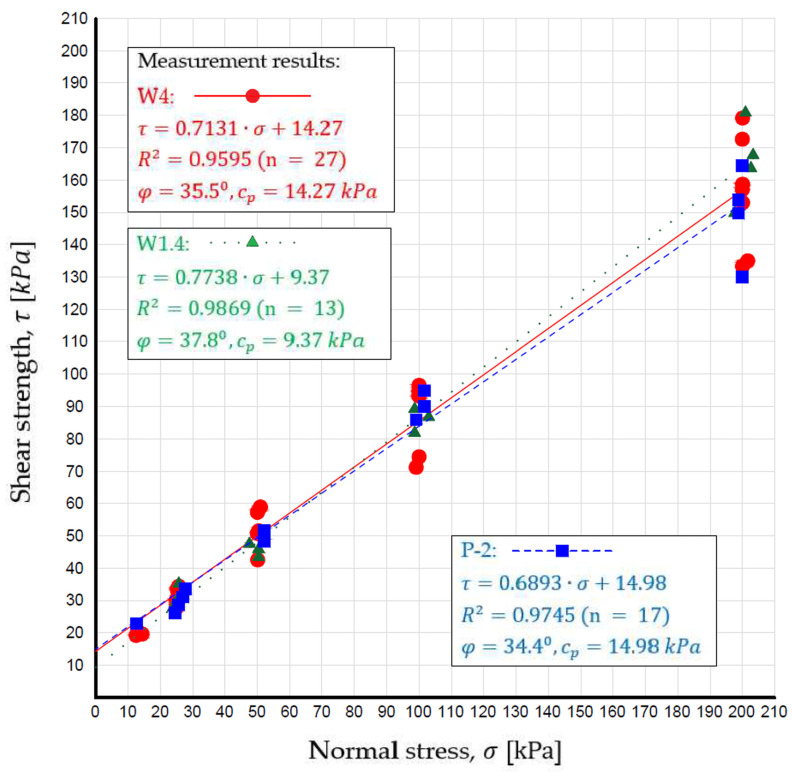
Direct shear test results for washed mineral waste.

Based on the direct shear test results, linear approximation equations were determined to represent the shear strength of WMW. Using these equations, the internal friction angle (φ) and apparent cohesion (c_p_) of the WMW were calculated and are presented in Figure 3 below the linear equations.

The internal friction angle values for WMW samples (W1.4 and P-2) are similar to those obtained by [40] for sample W4. The internal friction angle (φ) values for sample W1.4 fall within the characteristic range for coarse and medium sands in the dense and very dense state (37–39°). In the case of sample P-2, the values correspond to the range for fine and silty sands in the dense and very dense state (33–36°) as well as for coarse and medium sands in the medium-dense state (34–37°), as reported by [55].

The opinions on apparent cohesion in non-cohesive soils are divided. The article’s authors [82] claim that apparent cohesion does not exist in non-cohesive soils. On the other hand, other authors [83] indicate that capillary water in the pores of compacted non-cohesive soils may increase shear resistance due to the formation of additional soil cohesion (so-called apparent cohesion). The authors of [84] demonstrated apparent cohesion in fine sands due to capillary forces, although their experiment was limited to low normal stress levels not exceeding 1 kPa. Article [85] reported that apparent cohesion could reach values up to 16 kPa, depending on soil type, compaction degree, and degree of saturation. Additionally, as the degree of saturation increases, apparent cohesion initially rises, then stabilizes at a maximum value or begins to decrease. Studies by [86] confirm the presence of apparent cohesion in coarse-grained soils, reaching values of up to 26 kPa. However, as the degree of saturation increases, apparent cohesion values decrease to zero. In a soil sample with a gravel and sand fraction composition similar to the WMW samples, apparent cohesion disappeared at a degree of saturation of 0.35.

Due to the similar values obtained in the present study, the analysis of apparent cohesion in WMW samples was based on the research by [85]. The apparent cohesion value for sample P-2 was nearly identical to that of sample W4 [40], while the value for sample W1.4 was slightly lower. Considering the degree of saturation after compaction of WMW (0.63 and 0.68), the obtained cohesion values (9.37 and 14.98 kPa) for samples W1.4 and P-2, respectively, fall within the range of apparent cohesion values for dense medium sands (W1.4) and very dense fine sands (P-2).

**Figure 4:**
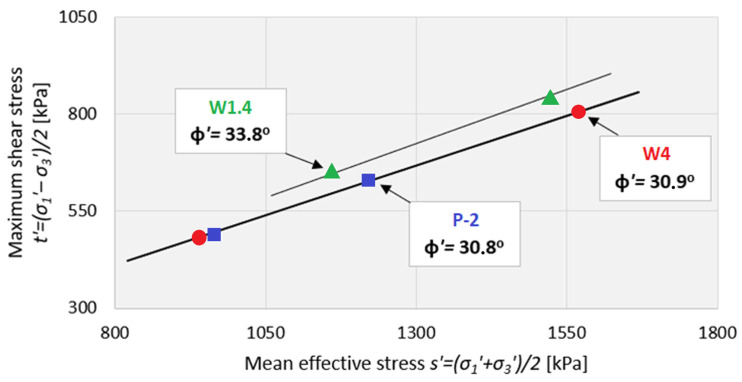
Triaxial shear test results (CIU type) for washed mineral waste.

In the case of the triaxial shear strength tests—isotropically consolidated undrained triaxial test—*CIU* type (Figure 4), the effective values of internal friction angle were determined according to classical methodology resulting from the Coulomb–Mohr theory [62]. For the representation and analysis of the results, standard MIT stress path parameters [56] have been used, i.e., mean effective stress (2) and shear stress (3), which is half of the deviatoric stress (q).

s′ = (σ′_1_ + σ_3_′)/2 (2)

t′ = (σ′_1_ − σ_3_′)/2 (3)

The maximum value of deviatoric stress was considered the shear stress criterion. The WMW samples contained sharp elements (e.g., glass and metals), and there were some ruptures due to using rubber membranes during testing. As a result, only two test results were presented for each sample. Therefore, these results are indicative rather than definitive. Nevertheless, the internal friction angle values obtained are comparable to those determined in the direct shear test.

The internal friction angle for the W1.4 specimen series was φ’ = 33.8°, the highest among the three analyzed triaxial compression test series. For the WMW samples from the P-2 series, the friction angle was φ’ = 30.8°, which was very close to the value obtained for the W4 series (φ’ = 30.9°). Due to the full water saturation stage preceding the triaxial compression tests, the apparent cohesion was negligible. The internal friction angles determined from the triaxial compression tests were slightly lower (by a few degrees) than those obtained from direct shear tests. This can be attributed to the unrestricted development of the shear plane in the triaxial test configuration.

**Figure 5:**
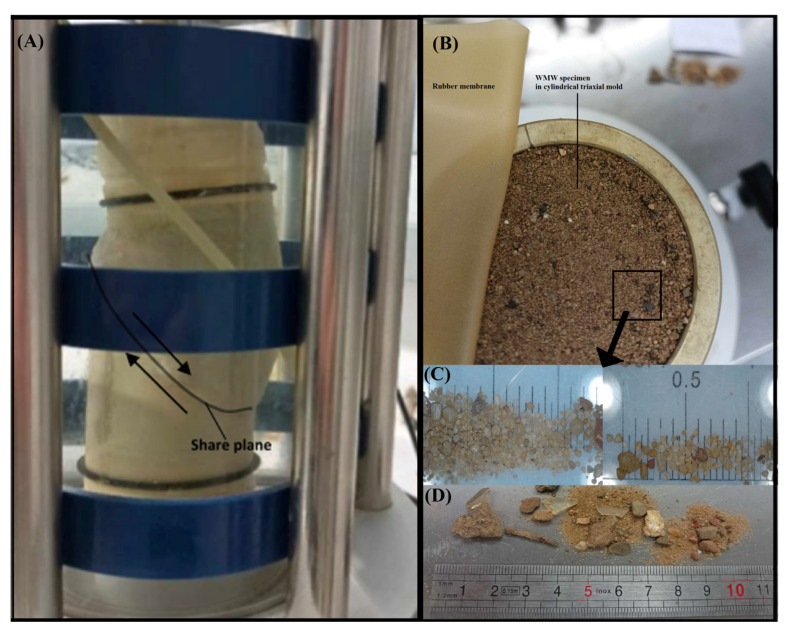
Sample of washed mineral waste: (**A**) in the shape of a sheared cylinder no. 1 (W4 series) in the chamber of the triaxial compression apparatus; (**B**) preparation for testing in the triaxial compression chamber—top view; (**C**) magnified image from an optical microscope; (**D**) sharp-edged anthropogenic materials found in waste (e.g., glass and metal) caused rubber membranes to crack during testing.

The dominant failure mode observed in the cylindrical samples was shear failure, characterized by developing a distinct shear plane. An example of such a specimen following the triaxial compression test is presented in Figure 5.

**Figure 6:**
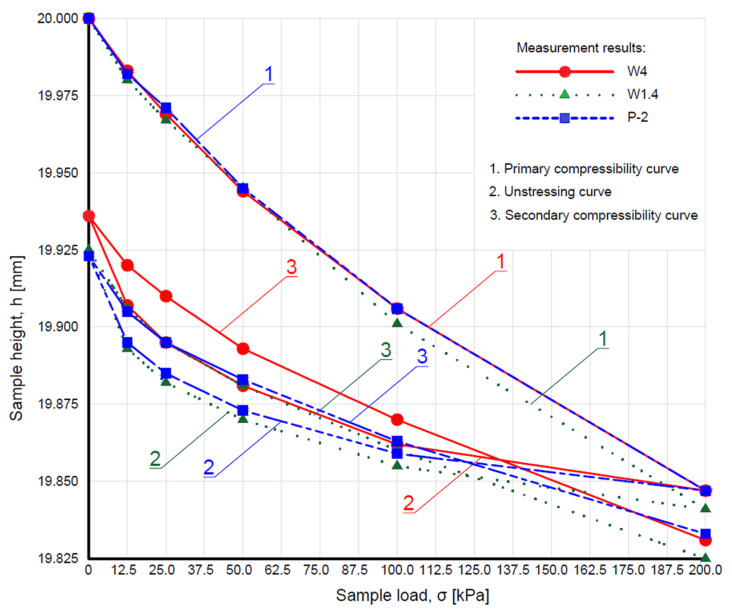
The compressibility and unstressing curves of washed mineral waste.

**Figure 7:**
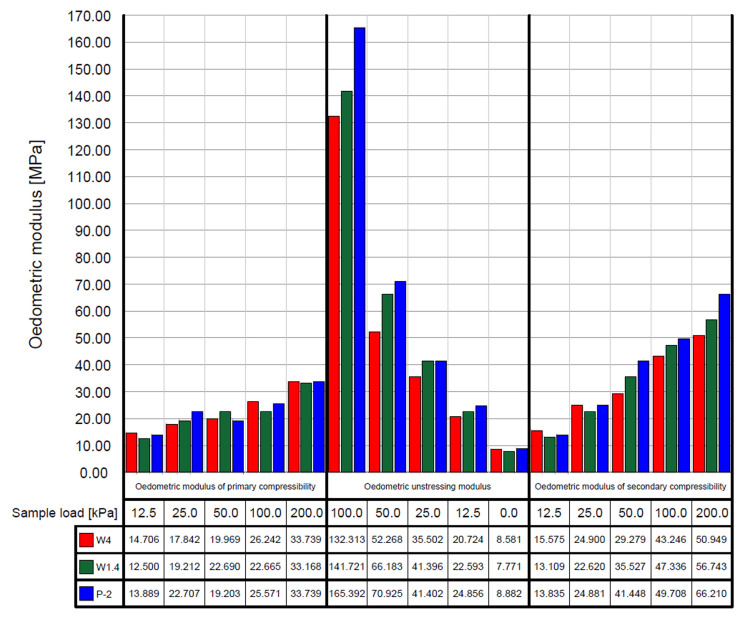
Average values of the oedometric modulus of washed mineral waste, depending on the applied load.

The compressibility and unstressing curves of WMW are presented in Figure 6. In contrast, the average values of the oedometric modulus of WMW, depending on the applied load, are shown in Figure 7.

The compressibility and unstressing curves of WMW exhibited a similar shape within the tested load range. All samples showed a comparable relationship for the primary compressibility curve (marked as no. 1 in Figure 6), which was reflected in the values of the oedometric modulus of primary compressibility (Figure 7), particularly noticeable in the highest load range (100–200 kPa). The most significant changes were observed during sample unloading in the 200–100 kPa range, where the values of the oedometric unstressing modulus differed by up to 30 kPa (20% of the value). The WMW was characterized by oedometric modulus of primary compressibility values mostly in the range of 15–30 MPa and oedometric modulus of secondary compressibility values between 15–50 MPa. The literature shows these values correspond to loose fine and silty sands [49].

### 3.3. Chemical Parameters

The results of chemical parameters are presented in Table 3.

The organic substance content in the WMW samples did not exceed 2%. The values presented in Table 3 fall outside the range defining soils as low-organic (2–6%) according to the European classification [78]. This classification indicates that WMW can be considered mineral soils containing organic matter.

The content of the organic substances obtained in the WMW is similar to the values reported in the literature (Table 4). The results do not exceed the value reported by [42] and the range presented by [37] for mineral waste. Additionally, the values align with the minimum levels reported by [39,41,43,45]. Furthermore, the organic substances content in the WMW was comparable to that obtained under laboratory conditions after the mineral waste’s cleaning and drying process [44].

The calcium carbonate content in the WMW samples ranged from 1 to 5%, allowing it to be classified as weakly calcareous soils [78]. Currently, the literature lacks direct references to the calcium carbonate content in WMW.

All tested samples of WMW exhibited similar zinc, copper, cadmium, chromium, nickel, and cobalt concentrations. The lowest average concentration of heavy metals in the WMW was found for cadmium (1.17–1.22 mg/kg d.m.), while the highest was for zinc (67.44–76.28 mg/kg d.m.), which is also reflected in another waste generated in wastewater treatment plants (sewage sludge) [87,88,89]. The maximum average concentrations of heavy metals in the WMW are as follows: Zn > Pb > Cu > Ni > Cr > Co > Cd (76.28 > 49.72 > 47.22 > 10.19 > 6.49 > 5.19 > 1.22 mg/kg d.m.).

Based on the literature data, a summary of heavy metal concentrations in both categories of mineral waste as material and in water extracts from mineral waste generated in wastewater treatment plants is presented in Table 5. So far, no results have been published demonstrating the concentrations of heavy metals in extracts from washed mineral waste.

Most samples of WMW exhibited higher concentrations of lead and cadmium, as well as slightly higher nickel content, compared to the maximum concentrations of WMW reported by [43]. However, the zinc, copper, and chromium concentrations in most samples are lower than the maximum values for this waste. When comparing the results with the mineral waste data presented by [37], the zinc and lead concentrations exceeded the reported range in most samples, whereas the concentrations of the remaining heavy metals were within the range presented by [37]. Differences in heavy metal concentrations between individual categories of waste may result from local conditions of technological processes in wastewater treatment plants.

The pH and specific electrical conductivity results of WMW are at a similar level for individual samples (W4, W1.4, P-2). The pH is slightly higher than the maximum pH value of mineral waste (6.6–7.3) reported by [41] and lower than the value for the sample classified as fine aggregate (8.3) in [37]. The pH value of washed mineral waste does not exceed the range of permissible values for substances particularly harmful to the aquatic environment (6.5–9.0) [90]. Currently, the literature lacks reference data on the specific electrical conductivity of WMW.

Zinc was not detected in all samples of aqueous extracts from WMW, nor were lead and chromium detected in samples W4 and W1.4. The smallest differences between maximum and minimum average concentrations were observed for cadmium and cobalt, while the most considerable differences were noted for copper and nickel. The maximum average concentrations of heavy metals in aqueous extracts are as follows: Cu > Pb > Ni > Co > Cd > Cr (1.48 > 1.36 > 0.43 > 0.24 > 0.10 > 0.04 mg/kg d.m.).

The concentrations of zinc, lead, cadmium, chromium, and nickel in all WMW extracts fall within the range of heavy metal concentrations in mineral waste reported by [42]. Only the copper concentration in sample W4 exceeds the permissible value. The concentrations of zinc, cadmium, chromium, and cobalt in the extracts of WMW were lower than those for other mineral waste [41]. However, the nickel concentration was higher in all analyzed samples, while lead (in sample P-2) and copper (in sample W4) exceeded the reported limits.

In all samples, a high level of heavy metal immobilization was achieved. The average immobilization levels of heavy metals are as follows: Zn > Cr > Pb > Cu > Ni > Co > Cd (100.00 > 99.79 > 99.09 > 98.79 > 97.51 > 95.83 > 92.72%). Cadmium was the least immobilized metal, while zinc was the most immobilized. Currently, the literature lacks references for the level of immobilization in WMW.

### 3.4. Assessment of the Suitability of Washed Mineral Waste as Materials for the Construction of Flood Embankments

The analysis of the test results of WMW and the assessment of their suitability as materials for the construction of flood embankments were based on Polish and international criteria for soil selection for the construction of flood embankment bodies, as well as regulations specifying the permissible content of substances in soil that pose a significant risk to surface land protection and the allowable limit leaching values of these substances.

The Polish set of criteria (Table 6) is based on standard describing the requirements and inspections for the acceptance of embankments forming water and land reclamation structures [91], technical conditions of execution and acceptance of earthworks [92], the regulation on technical conditions affecting the integrity of hydraulic structures and their management [93], as well as the initiation of assessment projects regarding the technical condition of flood embankments [94].

The international set of criteria (Table 7) includes recommendations for the location, design, and construction of small earth dams [95,96], the design of flood embankments [97], and roads and bridges [98].

According to [99], the Polish recommendations in Table 6 are general and primarily focused on identifying unsuitable soils for construction. Similar guidelines can be found in [96,97,98] based on specifying which soils should be avoided. Regarding [95], the fundamental principle for selecting soils for flood embankment construction is to prioritize local soils near the project site to minimize transportation costs while ensuring the safety of the designed structure.

None of the criteria presented in Table 6 and Table 7 directly describe using WMW to construct flood embankments. Regulation [93] allows for the use of anthropogenic soils in the construction of earth-retaining structures (dams, flood embankments, and canal levees), provided that the content of components subject to decomposition or dissolution in water does not threaten the durability and safety of the structure both during construction and operation. Authors [94] recognized anthropogenic soils as acceptable materials for constructing flood embankment bodies, provided that special requirements are met. However, they did not specify evaluation criteria or testing methods.

Washed mineral waste, when treated as embankment soils (sands, particularly medium sands), can be used to construct flood embankment bodies of all classes [94], provided that the required moisture and compaction parameters are met. For Class I–II structures, the compaction index, corresponding to a degree of compaction of at least 0.7, should be at least 0.97, whereas for Class III–IV structures, the compaction index, corresponding to a degree of compaction of at least 0.55, should be at least 0.95. The moisture content of the waste before compaction should be greater than 7.86–8.37% [91]. A similar classification of WMW as sands allows for their use in flood embankment construction, according to [96].

When embedding into structures, the specified waste should first be free of organic and anthropogenic contaminants and not be frozen [91,92,98].

Key criteria for verifying the suitability of the discussed waste for the construction of flood embankments include determining the content of organic substances, clay fraction, gypsum, and soluble salts, as well as the level of chemical contamination present in the waste, along with an assessment of its environmental impact according to separate regulations [91,92,94]. In addition to the properties listed in Polish recommendations, foreign criteria [96,98] indicate that weathered soils, carbonate soils, soils containing mica or shales, and those prone to spontaneous combustion should be avoided for the construction of flood embankments.

The WMW does not exceed the permissible values indicated in [91,92] regarding the organic substances and clay fraction content. Additionally, the calcium carbonate content classifies the waste as weakly calcareous soils, suggesting that it has a limited impact on the properties of the waste.

The permissible concentrations of substances posing significant risks to soil surface protection, as outlined in [100,101], depend on the soil depth, the soil group classified based on its use, the subgroup (if applicable) defined by its properties, and its hydraulic conductivity. Considering the potential application of washed mineral waste in flood embankments, the relevant soil groups for comparison with the study results are Group I (residential areas) and Group IV (transportation areas, including roads). A summary of the permissible heavy metal concentrations in the soil for both groups is provided in Table 8.

Regarding the release of heavy metals from waste materials that may be used as construction materials in the future, the concentrations of these substances in aqueous extracts were compared with the permissible leaching limits. These limits serve as criteria for waste acceptance at specific landfill types [102,103]. Table 8 presents the permissible leaching limits for heavy metals at a liquid-to-solid ratio of 10 L/kg d.m. for different landfill types.

The concentrations of all heavy metals in the WMW fall within the permissible limits for group IV soils. Lead is the only heavy metal exceeding the allowable limits for group I soils (sample P-2). The concentrations of all heavy metals in the remaining samples (W4 and W1.4) comply with the permissible limits for group I soils.

The concentration of heavy metals in aqueous extracts from WMW is significantly lower than the permissible leaching limits for hazardous waste landfills and landfills for non-hazardous waste. However, certain heavy metals limit the possibility of waste disposal in inert waste landfills. Specifically, cadmium, nickel, and lead exceed the permissible leaching limits for inert waste disposal. Cadmium exceeded the limit in all samples, while nickel and lead exceeded it in sample P-2.

## 4. Conclusions

In the context of developing economies and the increased demand for the fundamental resource sand, its alternative sources (within the Circular Economy framework) represent economically and environmentally friendly solutions that are globally recommended. However, there is a lack of studies comparing the characteristics of different types of waste concerning potential applications. The present study addresses the topic of washed mineral waste from a highly urbanized area (where many native natural deposits of sand and gravel have been depleted), namely the Warsaw metropolitan area. This waste was analyzed in the context of its potential use in flood embankments. Structures where such material could be utilized include hydro embankments, whose condition and quantity have deteriorated following the last regional flood event. As a result of a broad range of studies, a precise characterization of the analyzed waste was obtained. The values of the washed mineral waste’s physical, permeability, mechanical, and chemical parameters demonstrate consistency over time (similar values among samples collected in different years). It indicates a stable and durable technological process in the wastewater treatment plant. Based on the obtained laboratory test results and their analysis, the possibility of using the discussed waste as a material for flood embankment construction has been confirmed, provided that appropriate moisture and compaction parameters are ensured (the moisture content of the waste before compaction should exceed 7.86–8.37%, and the compaction index should be at least 0.97 for Class I–II structures and at least 0.95 for Class III–IV structures). Before embedding the waste into structures, it is necessary to remove organic and anthropogenic contaminants contained within or, in exceptional cases, to limit them to a level ensuring quality control of the waste. Additionally, chemical testing should be conducted to confirm its applicability. It is recommended to verify the obtained results through field observations and studies, which should include monitoring changes over time in the tested characteristics of the waste and changes in the condition of the structures where the material has been applied (e.g., settlement, surface soil displacement).

## Figures and Tables

**Table 1:** Sand recovered from mineral waste produced in wastewater treatment plants.

Name/Type of Material Indicated by the Authors	Conducted Tests	Suggested Applications	Site [References]
Sand from wastewater sewer cleaning (WS), with waste code 19 08 02	Humidity, bulk density, degradable organic substances, total organic carbon, total nitrogen, Na, Ca, K, Li, Ba, organic matter content (LOI), leachability of harmful substances, and heavy metals.	Soil-like materials.	Gliwice, Poland [39]
Washed mineral waste (WMW), with waste code 19 12 09	Organic matter content, granulometric composition (fraction content), sand equivalent, passive capillarity, specific density of solids, quantities characterizing the limiting states of compaction (dry density, void ratio, and porosity), maximum dry density, optimal moisture content, and degree of saturation after compaction, permeability coefficient, direct shear tests (internal friction angle and apparent cohesion).	Soil backfill and road embankment materials.	Warsaw, Poland [40]
Sand from sand separators of wastewater treatment plants, with waste code 19 08 02	Moisture, dry matter, bulk density, degradable organic substances, total organic carbon, ammonium nitrogen, grain size distribution, organic matter content (LOI), leachability of harmful substances, and heavy metals.	Construction aggregates.	Bielsko-Biala, Gliwice, Poland [41]
Sandy waste, with waste code 19 12 09	Fraction content, grain size distribution, and dry organic matter.	Alternative raw material in construction.	Warsaw, Poland [19]
Sand from grit chambers, with waste code 19 08 02	Dry matter, dry mineral matter, organic matter, grain-size composition, leachability of harmful substances and heavy metals, fluorides, and dissolved organic carbon.	Fine-grained aggregate in the production of concrete.	Cracow, Poland [42]
Waste from the grit chamber (GC) ^1^	Inorganic content, organic matter, and heavy metal content.	Building materials.	Shenzhen, China [37]
Waste sand, with waste code 19 12 09	Humidity, dry matter, dry mineral matter, dry organic matter, particle size distribution, content of harmful substances, and heavy metal.	Aggregate for the production of building materials (concrete), an additive to soils for backfilling excavations.	Mazovia, Poland [43]
Residual sand removed from grit chambers ^1^	Total solids, total fixed solids, total volatile solids, percentage of moisture, the composition of organic and mineral fractions retained in the sieves in the grain size test, axial compressive strength, and tensile strength by diametral compression of samples.	Fine aggregate in the preparation of non-structural concrete elements, e.g., sidewalks and curbs.	São Carlos, Brazil [44]
Sewage sand ^2^	Content of total solids, organic matter content (LOI), specific density, and particle size distribution.	Building materials.	Moravia, Czech Republic [45]

^1^ The inability to indicate the waste code is due to the location of the wastewater treatment plant. ^2^ The article was written before the establishment of the European Waste Catalogue in 2014 [38].

**Table 2:** Physical and permeability parameters of washed mineral waste.

No.	Parameter	Sample No.		
		W4 ^1^	W1.4	P-2
1.	Gravel fraction [%]	5.69 ^1^	9.42 ^1^	4.45
2.	Sand fraction [%]	94.17 ^1^	90.44 ^1^	95.43
3.	Silt + Clay fraction [%]	0.14 ^1^	0.14 ^1^	0.12
4.	Effective diameter D_60_ [mm]	0.48 ^1^	0.44 ^1^	0.55
5.	Effective diameter D_50_ [mm]	0.41 ^1^	0.40 ^1^	0.48
6.	Effective diameter D_30_ [mm]	0.31 ^1^	0.30 ^1^	0.35
7.	Effective diameter D_20_ [mm]	0.27 ^1^	0.24 ^1^	0.32
8.	Effective diameter D_10_ [mm]	0.22 ^1^	0.20 ^1^	0.25
9.	Uniformity coefficient C_U_ [−]	2.18 ^1^	2.20 ^1^	2.20
10.	Curvature coefficient C_C_ [−]	0.91 ^1^	1.02 ^1^	0.89
11.	Specific density ρ_s_ [g/cm^3^]	2.55 ^1^	2.62	2.62
12.	Dry density corresponding to the state of the loosest possible composition of soil grains ρ_dmin_ [g/cm^3^]	1.54 ^1^	1.56	1.57
13.	Maximum void ratio e_max_ [−]	0.656 ^1^	0.679	0.669
14.	Maximum porosity n_max_ [−]	0.396 ^1^	0.405	0.401
15.	Dry density corresponding to the state of the densest possible composition of soil grains ρ_dmax_ [g/cm^3^]	1.87 ^1^	1.84	1.85
16.	Minimum void ratio e_min_ [−]	0.364 ^1^	0.424	0.416
17.	Minimum porosity n_min_ [−]	0.267 ^1^	0.298	0.294
18.	Maximum dry density ρ_ds_ [g/cm^3^]	1.78 ^1^	1.78	1.79
19.	Optimum moisture content w_opt_ [%]	11.23 ^1^	11.34	11.95
20.	Degree of saturation after compaction S_r_ [−]	0.66 ^1^	0.63	0.68
21.	Hydraulic conductivity (in ITB-ZW-K2 apparatus) k_10_ [m/d] ^2^	5.37 ^1^	4.38	7.71
22.	Hydraulic conductivity (in triaxial compression apparatus) k_10_ [m/d] ^2^	2.6	0.9	1.1
23.	Hydraulic conductivity (empirical formulas) k10 [m/d]:	2406.77	2284.96	3361.87
	(a) Hazen formula	110.14	96.50	176.10
	(b) USBR formula	2.25 ^3^	3.07 ^3^	4.31 ^3^
	(c) Slichter formula	8.22 ^4^	8.40 ^4^	11.97 ^4^

^1^ Ref. [40]. ^2^ The tests were conducted on samples compacted to the parameters obtained during the compaction test in the Proctor apparatus (No. 18–19). ^3^ The result obtained from the Slichter formula for the minimum porosity value n_min_. ^4^ The result obtained from the Slichter formula for the maximum porosity value n_max_.

**Table 3:** Chemical parameters of washed mineral waste ^1^.

No.	Parameter	Sample no.		
		W4	W1.4	P-2
1.	Organic substances content [%]	1.49 ± 0.14	0.24 ± 0.01	1.01 ± 0.14
2.	Calcium carbonate content [%]	2.63 ± 0.63	2.25 ± 0.94	3.29 ± 0.91
3.	pH [−]	7.68–7.81	7.36–7.61	7.36–7.50
4.	Specific electrical conductivity [mS/cm]	0.260 ± 0.016	0.369 ± 0.061	0.421 ± 0.012
5.	Zinc (Zn) content: - in material [mg/kg d.m.] - in extract [mg/kg d.m.] - immobilization level [%]	67.44 ± 13.46 n.d. ^2^ ~100	72.32 ± 26.55 n.d. ^2^ ~100	76.28 ± 21.90 n.d. ^2^ ~100
6.	Lead (Pb) content: - in material [mg/kg d.m.] - in extract [mg/kg d.m.] - immobilization level [%]	14.12 ± 0.12 n.d. ^2^ ~100	31.34 ± 34.36 n.d. ^2^ ~100	49.72 ± 81.56 1.36 ± 0.01 97.20
7.	Copper (Cu) content: - in material [mg/kg d.m.] - in extract [mg/kg d.m.] - immobilization level [%]	47.22 ± 17.35 1.48 ± 0.12 96.87	38.44 ± 12.44 0.03 ± 0.02 99.92	29.13 ± 22.29 0.12 ± 0.09 99.59
8.	Cadmium (Cd) content: - in material [mg/kg d.m.] - in extract [mg/kg d.m.] - immobilization level [%]	1.22 ± 0.29 0.08 ± 0.01 93.44	1.17 ± 0.15 0.10 ± 0.02 91.45	1.19 ± 0.17 0.08 ± 0.03 93.28
9.	Chromium (Cr) content: - in material [mg/kg d.m.] - in extract [mg/kg d.m.] - immobilization level [%]	5.84 ± 2.61 n.d. ^2^ ~100	3.79 ± 0.74 n.d. ^2^ ~100	6.49 ± 3.26 0.04 ± 0.01 99.38
10.	Nickel (Ni) content: - in material [mg/kg d.m.] - in extract [mg/kg d.m.] - immobilization level [%]	10.19 ± 0.87 0.21 ± 0.06 97.94	9.58 ± 3.19 0.11 ± 0.05 98.85	10.07 ± 1.46 0.43 ± 0.11 95.73
11.	Cobalt (Co) content: - in material [mg/kg d.m.] - in extract [mg/kg d.m.] - immobilization level [%]	4.98 ± 0.58 0.20 ± 0.05 95.98	5.19 ± 2.29 0.17 ± 0.04 96.72	4.61 ± 0.72 0.24 ± 0.03 94.79

^1^ The average concentration of heavy metals in WMW and water extracts from WMW in [mg/dm^3^] are presented in the Supplementary Materials, Tables S1 and S2, respectively. ^2^ n.d.—not detected.

**Table 4:** The organic matter content in mineral waste generated in wastewater treatment plants [%].

Type of Waste	Value	References
Washed mineral waste	0.78–14.44	[43]
Mineral waste	5.50–8.00 ^1^	[37]
	1.11	[39]
	1.11–15.91	[41]
	1.80 ^2^	[42]
	1.00 ± 0.17 ^3^	[44]
	1.58–68.49	[45]

^1^ Samples containing a predominant sand fraction labeled as GC (grit chamber waste)—LWPP (lightweight aggregate). ^2^ Mineral waste samples from grit chambers. ^3^ Mineral waste from the grit chamber after cleaning and drying under laboratory conditions.

**Table 5:** Heavy metal content in material and water extracts of mineral waste generated in wastewater treatment plants.

Heavy Metal Content	In material		In extracts		
Unit	[mg/kg d.m.]		[mg/dm^3^]		[mg/kg d.m.]
Metal/Type of Waste	WMW ^1^	MW ^2^	MW ^3^	MW ^4^	
Zn	<5.0–491	69.75–73.50	<0.08	0.10–0.35	1.00–3.50
Pb	<5.0–17.5	20.25–49.00	<0.20	0.005–0.05	0.05–0.50
Cu	<5.0–677	10.50–81.00	<0.06	n.d. ^6^—0.08	n.d. ^6^—0.80
Cd	<0.5	6.75–10.50	<0.04	n.d. ^6^—0.02	n.d. ^6^—0.20
Cr	<5.0–11	0.00–10.50	<0.20	0.02–0.04	0.20–0.40
Ni	<5.0–9.76	3.50–24.75	<0.20	n.d. ^6^	n.d. ^6^
Co	– ^5^	– ^5^	– ^5^	n.d. ^6^—0.04	n.d. ^6^—0.40

^1^ Washed mineral waste [43]. ^2^ Mineral waste [37]: approximate values derived from LWPP (fine aggregates) and GC (grit chamber waste). ^3^ Mineral waste [42]. ^4^ Mineral waste [41]. ^5^ Not analyzed by the authors of the papers. ^6^ Not detected.

**Table 6:** Polish criteria for selecting soils for the construction of flood embankment bodies.

Guidelines	PN-B-12095:1997 Standard [91]	Technical Conditions of Execution and Acceptance of Earthworks [92]	Assessment Projects Regarding the Technical Condition of Flood Embankments [94]
Acceptable soils:	Organic (excluding certain peats and gyttjas) for class ^1^ III–IV structures.	No data	Mineral, organic, and anthropogenic.
Indicative scope and conditions of use:	Moisture content before compaction in non-cohesive soils should be greater than 0.7w_opt_. Embankment compaction in non-cohesive soils: (a) fine and medium sands: - Class I–II: I_D_ ≥ 0.7, - Class III–IV: I_D_ ≥ 0.55, (b) coarse sands and coarse-grained soils: - Class I–II: ID ≥ 0.65, - Class III–IV: ID ≥ 0.55.	Moisture content before compaction within the range of w_opt_ ± 2% (specified only for cores and screens of earth dams).	Mineral soils: (a) coarse-grained and fine-grained non-cohesive soils (gravels, tills, sands) are suitable for embankment bodies of all classes; Organic soils may be used for embankment construction under appropriate supervision ^2^ when mineral soils are unavailable or on organic subsoil (certain types of peat are not recommended); Anthropogenic soils may be used after meeting special requirements ^2^; Additionally, all materials must comply with the moisture and compaction criteria specified in the standard [91].
Soils should not contain:	Frozen soils, wastes, rubble, plant debris, tree stumps, and other contaminants whose quality cannot be controlled.	Frozen soils, soils whose quality cannot be controlled, and soils containing contaminants (wastes, rubble, plant debris, tree stumps).	No data
Unsuitable soils:	With an organic substance content exceeding 2%; With a clay fraction content exceeding 30%; Containing gypsum and soluble salts ^2^; Chemically contaminated ^2^.	With an organic substance content exceeding 3%; With a clay fraction content exceeding 30%; Containing gypsum and soluble salts exceeding 15%; Chemically contaminated ^2^.	Swelling and water-soluble (incorporated without special treatments). ^2^

^1^ Regulation [93] presents the classification of major hydraulic structures. The classes of structures refer to their name, nature, or function, as well as the values of indicators resulting from the size of the structure. ^2^ No evaluation criteria or testing methods have been specified.

**Table 7:** International criteria for selecting soils for the construction of flood embankment bodies.

Guidelines	Manual on Small Earth Dams [96]	Design of Small Dams [95]	The International Levee Handbook [97]	Design Manual for Roads and Bridges [98]
Most recommended soils:	Sands and clays, as well as a mixture of both fractions.	Locally available (found as close to the construction site as possible), allowing for proper design and ensuring the structure’s safety; Coarse-grained sands and gravels are the most suitable. Fine sands, silts, and silty soils (use with caution).	No data	No data
Not recommended soils:	No data	Organic (due to their susceptibility to decomposition).	No data	No data
Soils to avoid:	Organic; Weathering (decomposing); With a high mica content (which creates a slip surface in soils with a low clay fraction content); Carbonate; Metamorphic and sedimentary shales (prone to disintegration when soaked; also containing mica).	No data	Organic (with a significant organic substances content).	Containing branches, roots, stumps, wood, or plastic waste; Frozen; Prone to spontaneous combustion; Swelling or collapsible; Contaminated with harmful chemical substances.

**Table 8:** Permissible contents of substances causing risk, divided into groups and subgroups of soils [100,101] along with leaching limits values of heavy metals at a liquid-to-solid ratio L/S = 10 L/kg d.m. at landfills of a given type [102,103], in [mg/kg d.m.].

Permissible Contents of Substances Causing Risk [mg/kg d.m.]								
Group of Soil	For Depth Below Ground Level [m]	Zn	Pb	Cu	Cd	Cr	Ni	Co
I ^1^	0–0.25	500	200	200	2	200	150	50
	>0.25 ^3^	300	100	150	3	300	100	30
IV ^2^	0–0.25	2000	600	600	15	1000	500	200
	>0.25 ^3^	300	200	200	6	300	100	50
**Leaching limits of heavy metals at landfills of a given type [mg/kg d.m.]**								
Hazardous waste		200	50	100	5	70	40	– ^4^
Non-hazardous waste		50	10	50	1	10	10	– ^4^
Inert waste		4	0.5	2	0.04	0.5	0.4	– ^4^

^1^ Residential development area. ^2^ Communication area. ^3^ Hydraulic conductivity value higher than or equal to 1 × 10^−7^ m/s. ^4^ Not included in [102,103].

## Data Availability

The raw data supporting the conclusions of this article will be made available by the authors on request.

## Supplementary Materials

The following supporting information can be downloaded at: https://www.mdpi.com/article/10.3390/ma18143384/s1, Table S1: The average content of heavy metals (Zn, Pb, Cu, Cd, Cr, Ni, and Co) in washed mineral waste [mg/dm^3^]; Table S2: The average content of heavy metals (Zn, Pb, Cu, Cd, Cr, Ni, and Co) in water extracts from washed mineral waste [mg/dm^3^].

## Abbreviations

The following abbreviations are used in this manuscript:

Ba	Barium
Ca	Calcium
Cd	Cadmium
CE	Circular economy
CIU	Isotropically consolidated undrained triaxial test
Co	Cobalt
Cr	Chromium
Cu	Copper
d.m.	Dry matter
FAAS	Flame Atomic Absorption Spectrometry
GC	Grit chamber
K	Potassium
Li	Lithium
LOI	Loss on ignition
L/S	Liquid-to-solid phase ratio
LWPP	Luofang Water Purification Plant
MIT	Massachusetts Institute of Technology
MPS	Microwave Preparation System
MW	Untreated mineral waste obtained directly from grit chambers
Na	Sodium
Ni	Nickel
Pb	Lead
PE	Population equivalent
USBR	United States Bureau of Reclamation formula
WMW	Washed mineral waste received from the rinsing process of the mineral waste from grit chambers
WS	Sand from wastewater sewer cleaning
Zn	Zinc
