# Supplementary material for: Potential Use of Wastewater Treatment Plant Washed Mineral Waste as Flood Embankment Materials
Source: Materials (Basel). 2025 Jul 18;18(14):3384. doi: 10.3390/ma18143384 (PMC12298621; doi:10.3390/ma18143384)
Supplement: Supplementary file 1 [file materials-18-03384-s001.zip › materials-3734841-supplementary.pdf]

Table S1: The average content of heavy metals (Zn, Pb, Cu, Cd, Cr, Ni, and Co) in washed mineral waste [mg/dm^3].

Sample no.	Zn	Pb	Cu	Cd	Cr	Ni	Co
W4	1.544±0.305	0.323±0.001	1.081±0.396	0.028±0.007	0.134±0.061	0.233±0.022	0.114±0.014
W1.4	1.661±0.606	0.718±0.784	0.884±0.288	0.027±0.003	0.087±0.017	0.220±0.074	0.119±0.053
P-2	1.882±0.758	1.180±1.880	0.699±0.510	0.029±0.005	0.159±0.084	0.243±0.044	0.111±0.018

Table S2: The average content of heavy metals (Zn, Pb, Cu, Cd, Cr, Ni, and Co) in water extracts from washed mineral waste [mg/dm^3].

Sample no.	Zn	Pb	Cu	Cd	Cr	Ni	Co
W4	n.d.	n.d.	0.148±0.012	0.008±0.001	n.d.	0.021±0.006	0.020±0.005
W1.4	n.d.	n.d.	0.003±0.002	0.010±0.002	n.d.	0.011±0.005	0.017±0.004
P-2	n.d.	0.136±0.001	0.012±0.009	0.008±0.003	0.004±0.001	0.043±0.011	0.024±0.003

n.d. – not detected
